# “Permissiveness, guiltiness, anxiety”: A qualitative study on emotional meanings of school task procrastination reported by occupational therapy students in South-eastern Brazil

**DOI:** 10.1192/j.eurpsy.2022.1788

**Published:** 2022-09-01

**Authors:** E. Turato, E. Santos

**Affiliations:** State University of Campinas, Laboratory Of Clinical-qualitative Research, Campinas, Brazil

**Keywords:** mental health care, Qualitative research, procrastination, Occupational therapy

## Abstract

**Introduction:**

According to the Medical Subject Headings, the vocabulary used by PubMed, procrastination is ‘the deferment of actions or tasks to a later time, or to infinity’. Studies on procrastination are increasing, especially among university students, gaining prominence in academic literature. However, studies on the procrastination phenomenon have been mainly quantitative, correlating such experiences with clinical and behavioral manifestations. Specific research with occupational therapy students is lacking in the literature.

**Objectives:**

To interpret symbolic meanings related to life experiences of the procrastination phenomenon of school tasks as reported by occupational therapy undergraduate students, self-referred as procrastinators.

**Methods:**

Clinical-qualitative design. Data collected through semi-directed interviews with open-ended questions in-depth. Clinical-Qualitative Content Analysis generated categories discussed in the light of the psychodynamic theoretical framework. This study was carried out in a private Brazilian university. The sample was closed by the information saturation criterion.

**Results:**

Seven students were interviewed. Procrastination comes associated with anxiety as productivity, but not reported as an “executive drive”, that would imprison the individual in a vicious cycle. There are defense mechanisms referred to as self-preservation for not assume responsibilities for tasks. Ineffective strategies seem to be experienced by the students to avoid procrastination, but without resolving possible psychodynamic conflicts related to the task.

**Conclusions:**

Students’ procrastination ambivalently affects their daily lives, although they can report the phenomenon as negative. It is suggested further qualitative studies that explore specifically meanings of procrastinating personal activities, in general, considering these individuals will work precisely in a therapeutic approach in the field of occupations of the people.

**Disclosure:**

No significant relationships.

